# Low serum neurofilament light chain values identify optimal responders to dimethyl fumarate in multiple sclerosis treatment

**DOI:** 10.1038/s41598-021-88624-7

**Published:** 2021-04-29

**Authors:** Paulette Esperanza Walo-Delgado, Susana Sainz de la Maza, Noelia Villarrubia, Enric Monreal, Silvia Medina, Mercedes Espiño, José Ignacio Fernández-Velasco, Eulalia Rodríguez-Martín, Ernesto Roldán, Daniel Lourido, Alfonso Muriel, Jaime Masjuan-Vallejo, Lucienne Costa-Frossard, Luisa María Villar

**Affiliations:** 1 Immunology Department, Ramón y Cajal University Hospital, IRYCIS, REEM, Ctra. Colmenar Km. 9.100, 28034 Madrid, Spain; 2 Neurology Department, Ramón y Cajal University Hospital, IRYCIS, REEM, Madrid, Spain; 3 Radiology Department, Ramón y Cajal University Hospital, IRYCIS, REEM, Madrid, Spain; 4 Clinical Biostatistics Unit, Ramón y Cajal University Hospital, IRYCIS, University of Alcalá, CIBERESP, Madrid, Spain

**Keywords:** Immunology, Autoimmunity, Immunological disorders, Lymphocytes, Neuroimmunology, Translational immunology, Neuroscience, Diseases of the nervous system, Molecular neuroscience, Neuroimmunology, Neurology, Multiple sclerosis

## Abstract

Serum neurofilament light chains (sNfL) are biomarkers of disease activity in multiple sclerosis (MS), but their value to predict response to treatment, and their association with patient immunological profile, need to be further explored. We studied 80 relapsing–remitting MS patients initiating dimethyl fumarate (DMF) treatment. sNfL levels were explored at baseline and at 3, 6 and 12 months by single molecule array. Blood lymphocyte subsets were measured at baseline and at 6 months by flow cytometry. Patients were followed a year and classified as NEDA (no evidence of disease activity) or ODA (ongoing disease activity). NEDA patients had lower sNfL levels at baseline (*p* = 0.0001), and after three (*p* = 0.004) and six (*p* = 0.03) months of DMF treatment. Consequently, low baseline sNfL values (≤ 12 pg/ml) increased the probability of NEDA (OR 5.8; CI 1.82–15.6; *p* = 0.002, after correcting by disease activity in the previous year), and associated with significant reductions of central memory CD4+ T lymphocytes, interferon-gamma+ CD8+ T lymphocytes, Natural Killer T cells, and memory B cells upon DMF treatment, being the highest differences in memory B cells (*p* < 0.0001). This shows that low baseline sNfL values identify MS patients with higher probability of optimal response to DMF and of a reduction in effector immune cells.

## Introduction

Dimethyl fumarate (DMF; Biogen, Cambridge, MA), is an approved treatment for relapsing remitting multiple sclerosis (RRMS). DMF demonstrated efficacy in ameliorating disease course in RRMS patients in two large 2-year double-blind, multinational, phase III trials, DEFINE^1^ and CONFIRM^2^ and their dose-blind extension, ENDORSE^3,4^. Optimal responders to this drug show a shift from an inflammatory to a tolerogenic blood cell profile^5^. Serum neurofilament light chains (sNfL) are biomarkers of inflammation and axonal damage in MS, and several studies support their role as biomarkers of treatment response^6–11^. Results from a prospective open label phase IV trial TREMEND showed that DMF was effective in reducing NfL concentration in CSF, plasma and serum of treatment-naïve RRMS patients and that NfL levels in the CSF at 12 months of DMF treatment associated with disease activity^12^. In another study performed in naïve and previously treated RRMS patients, the ability of DMF in decreasing sNfL levels was similar to that of Teriflunomide but relatively lower than high efficacy drugs as Fingolimod, Natalizumab or Alemtuzumab, and was highly dependent on baseline sNfL levels^13^.

The aim of this work was to study sNfL changes during DMF treatment and to explore if they could be early predictors of treatment response in MS.

## Results

### DMF treatment decreased sNfL values in RRMS patients

We included 80 RRMS patients (76% females) who initiated consecutively treatment with DMF in this prospective longitudinal study. Baseline clinical and demographic characteristics are shown in Table 1. Patient age was 41.5 (33.6–47.3) years (median, IQR), disease duration was 9.5 (4.4–16.3) years and EDSS was 1.5 (1.5–2.5). Fifty five patients (68.7%) received previous disease modifying treatments.

**Table 1:** Baseline clinical and demographic data of patients included in the study.

Variable	(n = 80)
Age (years). Median (IQR)	41.5 (33.6–47.3)
Disease duration (years). Median (IQR)	9.5 (4.4–16.3)
Sex (Male/female)	19/61
EDSS score. Median (IQR)	1.5 (1.5–2.5)
MSSS score. Median (IQR)	2.2 (1.3–4.2)
N. of total previous relapses. Median (IQR)	4 (2–6)
N. of patients with relapses in the previous year (Yes/no)	50/30
N. of relapses in the previous year. Median (IQR)	1 (0–1)
Time from the last relapse (months). Median (IQR)	6.0 (3.0–9.2)
N. of patients with relapses in the previous 3 months (Yes/no)	16/64
N. of T2 lesions. Median (IQR)	19 (12–27)
N. of patients with Gd+ enhanced lesions (Yes/no)	24/56
N. of Gd+ enhanced lesions. Median (IQR)	0 (0–1)
Previous treatments (Naïve/Previously treated)	25/55
Previous treatments (FL/SL)	45/10
Washout period (months). Median (IQR)	2 (2–4)

*EDSS* Expanded Disability Status Scale, *Gd* gadolinium, *FL* first line drugs, *IQR* interquartile range, *MSSS* Multiple Sclerosis Severity Score, *N.* number, *SL* second line drugs.

**Figure 1:**
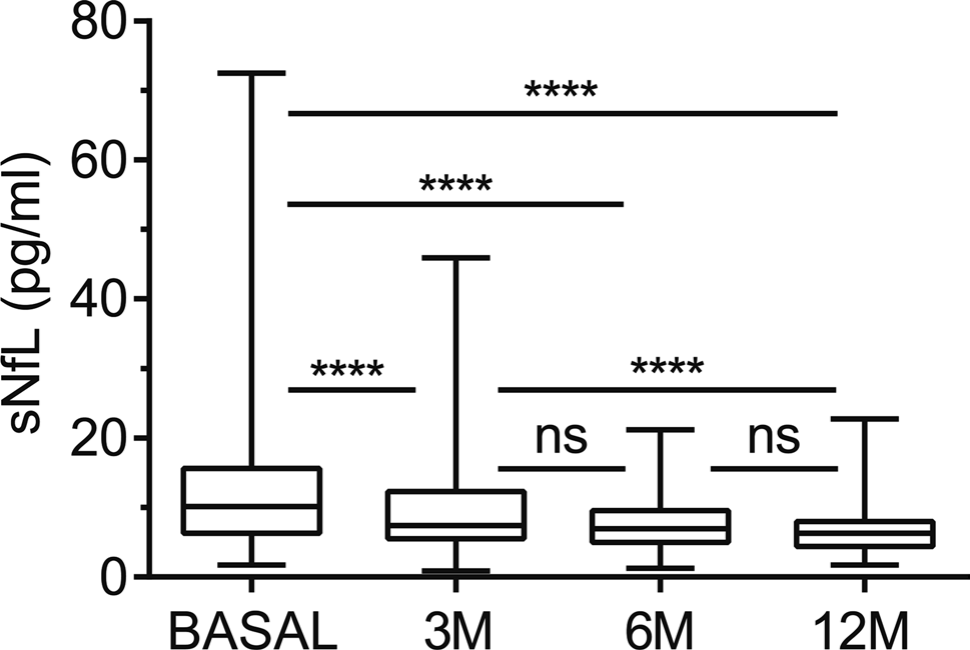
Serum neurofilament light chain (sNfL) levels in relapsing–remitting multiple sclerosis (RRMS) patients. sNfL concentration (median, IQR) obtained in 80 RRMS patients at baseline (Basal) and 3, 6, and 12 months (M) after dimethyl fumarate initiation. ****p < 0.0001; *ns* not significant.

Median (IQR) baseline sNfL concentration was 10.1 (6.3–15.6) pg/ml. We observed a clear decline in sNfL values during the first year of DMF treatment (Fig. 1). It became evident at 3 months (median decrease from baseline: 4.0 pg/ml; CI 2.4–5.6; *p* < 0.0001), being less pronounced from 3 to 12 months of treatment, when median sNfL decrease from baseline was 6.7 pg/ml (CI 5.5–8.3; *p* < 0.0001).

To investigate the influence of disease activity at baseline in sNfL decrease, we classified patients according to baseline disease activity as “Active Disease” (AD, n = 30) or “No Active Disease” (NAD, n = 50). AD patients were defined as those showing Gd+ enhancing lesions in the basal MRI or having relapses in the previous three months to treatment initiation. AD patients had a modest increase in sNfL levels at baseline compared to NAD ones (*p* = 0.04), and this difference was lost after three months of DMF treatment. In addition, both groups of patients experienced a clear decrease in sNfL values during the first year of treatment, independently of previous disease activity (*p* < 0.0001 in both cases).

### Association of sNfL values with response to DMF

We first explored baseline clinical and demographic differences between patients achieving NEDA (n = 50, 62.5%) or ODA (n = 30, 37.5%) status at 12 months of DMF treatment. As shown in Table 2, NEDA patients had lower numbers of Gd+ enhancing lesions (*p* = 0.006). Likewise, we observed a moderate decrease in the proportion of patients showing Gd+ enhancing lesions at baseline in the same group (*p* = 0.02). No differences were found in other clinical, demographic or radiological variables.

**Table 2:** Baseline clinical and demographic data of NEDA and ODA patients.

Variable	NEDA (n = 50)	ODA (n = 30)	*p*
Age (years)	42.2 (36.3–47.8)	38.2 (28.3–46.5)	ns
Disease duration (years). Median (IQR)	9.9 (5.9–16.7)	7.7 (2.2–13.3)	ns
Sex (male/female)	13/37	6/24	ns
EDSS score. Median (IQR)	1.5 (1.5–2.5)	1.5 (1.5–3.0)	ns
MSSS score. Median (IQR)	2.1 (1.2–4.5)	2.4 (1.8–3.3)	ns
N. of previous relapses. Median (IQR)	4 (2–5)	4 (2–7)	ns
Patients with relapses in the previous year (Yes/No)	28/22	22/8	ns
N. of relapses in the previous year. Median (IQR)	1 (0–1)	1 (0–1)	ns
Time from the last relapse (months). Median (IQR)	6.0 (3.0–9.7)	4.5 (3.0–9.5)	ns
Patients with relapses in the previous 3 months (Yes/No)	8/42	8/22	ns
N. of T2 lesions. Median (IQR)	18 (11–26)	19 (11–37)	ns
Patients with Gd + enhancing lesions (Yes/No)	10/40	14/16	0.02
N. of Gd+ enhancing lesions. Median (IQR)	0 (0–0)	0 (0––2)	0.006
Treatment naïve/ Previously treated	17/33	8/22	ns
Previous treatments (FL/SL)	25/8	20/2	ns
Wash-out period (months). Median (IQR)	2 (2–4)	3.0 (1.7–5.5)	ns

*EDSS* Expanded Disability Status Scale, *Gd* Gadolinium, *FL* first line drugs, *IQR* interquartile range, *NEDA* no evidence of disease activity, *MSSS* Multiple Sclerosis Severity Score, *N.* number, *NS* NOT significant, *ODA* ongoing disease activity, *SL* second line drugs.

**Figure 2:**
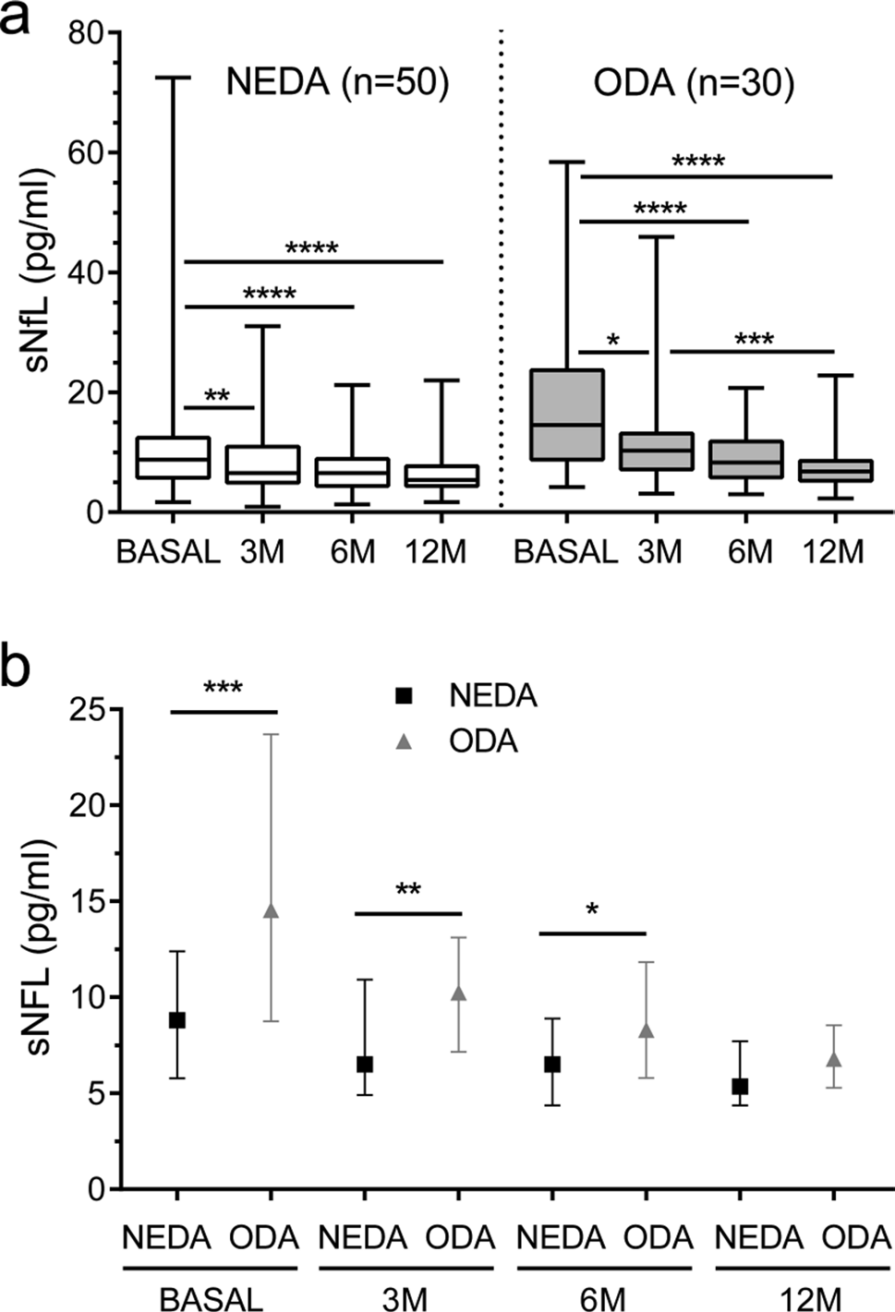
Serum neurofilament light chain (sNfL) values in NEDA and ODA patients. (**a**) sNfL levels (median, IQR) of NEDA (white rectangle; n = 50) and ODA (grey rectangle; n = 30) relapsing–remitting multiple sclerosis patients at baseline (basal) and at 3, 6, and 12 months (M) of dimethyl fumarate treatment initiation. (**b**) Differences in sNfL values (median, IQR) between NEDA (black square) and ODA (grey triangle) patients at each time point. Only *p* values below 0.05 are shown. *p < 0.05; **p < 0.01; ***p < 0.001; ****p < 0.0001. *NEDA* no evidence of disease activity, *ODA* ongoing disease activity.

We further explored differences in baseline, 3, 6 and 12-months sNfL values between patients achieving NEDA or ODA status at 1 year of treatment. NEDA and ODA patients experienced a gradual decrease in sNfL values during the first year of DMF treatment (*p* < 0.0001 in both cases). However, sNfL stabilization occurred at three months of DMF treatment in NEDA patients, while it took 12 months in ODA ones (Fig. 2a). In addition, NEDA patients had lower sNfL levels at baseline (Median [IQR]: 8.8 [5.8–12.4] vs 14.5 [8.7–23.7] pg/ml; *p* = 0.0001), and at three (6.5 [4.9–10.9] vs 10.2 [7.1–13.1] pg/ml; *p* = 0.004) and six months (6.5 [4.4–8.9] vs 8.3 [5.8–11.8] pg/ml; *p* = 0.03) of DMF treatment than ODA ones, although differences were lost after 12 months (5.3 [4.4–7.7] vs 6.8 [5.3–8.5] pg/ml; *p* = 0.150) (Fig. 2b).

**Figure 3:**
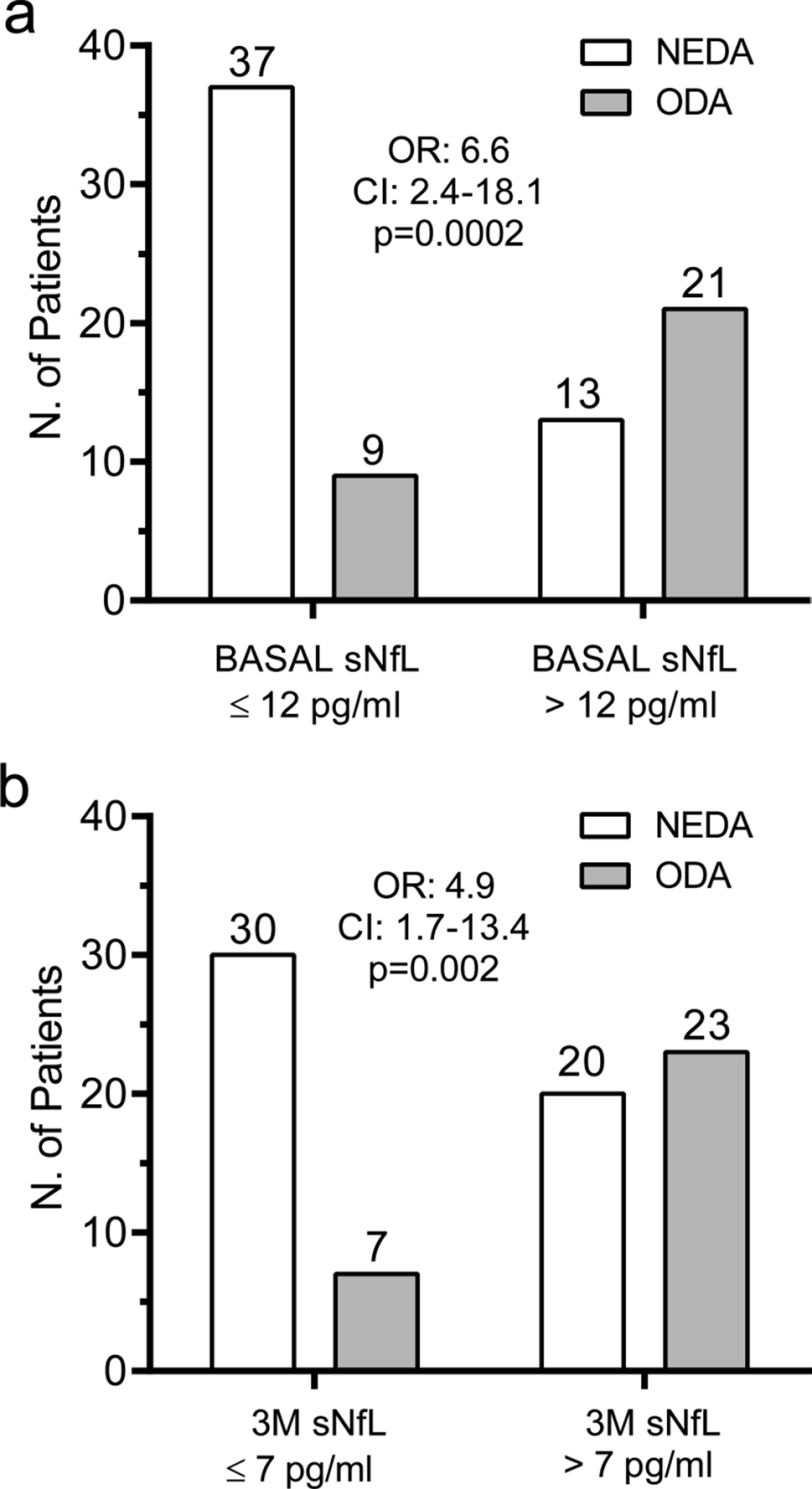
Serum neurofilament light chain (sNfL) values identify optimal responders to dimethyl fumarate (DMF) treatment. Number (N.) of relapsing–remitting multiple sclerosis patients achieving NEDA (white rectangle) or ODA (grey rectangle) status after 12 months of DMF treatment, depending on their baseline (basal) sNfL (**a**) or their 3-months (M) sNfL levels (**b**). *CI* 95% confidence interval, *NEDA* no evidence of disease activity, *ODA* ongoing disease activity, *OR* odds ratio.

We next studied if baseline and 3-months sNfL values could predict response to DMF treatment. Using ROC curves, we established a cut-off value of 12.0 pg/ml for baseline sNfL levels (AUC: 0.75; CI 0.63–0.86; *p* = 0.0002). As shown in Fig. 3a, patients showing baseline sNfL below 12 pg/ml had increased probability to achieve NEDA status at 12 months (OR 6.6; CI 2.4–18.1; *p* = 0.0002). After performing a multivariate analysis and adjusting results by presence of baseline Gd+ enhancing lesions, number of Gd+ enhancing lesions and of NEDA status in the previous year, we found that sNfL ≤ 12 pg/ml at baseline maintained its ability in predicting NEDA at 12 months (OR 5.8; CI 1.8–15.6; *p* = 0.002).

ROC curves also let us to establish a cut-off value of sNfL values of 7 pg/ml at three months of DMF treatment (AUC: 0.69; CI 0.57–0.81; *p* = 0.004). Figure 3b shows that patients with sNfL below 7 pg/ml at three months showed higher probability of being NEDA at 12 months (OR 4.9; 1.8–13.6; *p* = 0.002). Adjusting by presence and number of baseline Gd+ enhancing lesions and by NEDA status in the previous year, the OR obtained was 4.8 (CI 1.6–14.2; *p* = 0.005).

### Association between sNfL values and changes in blood leukocyte subsets

**Figure 4:**
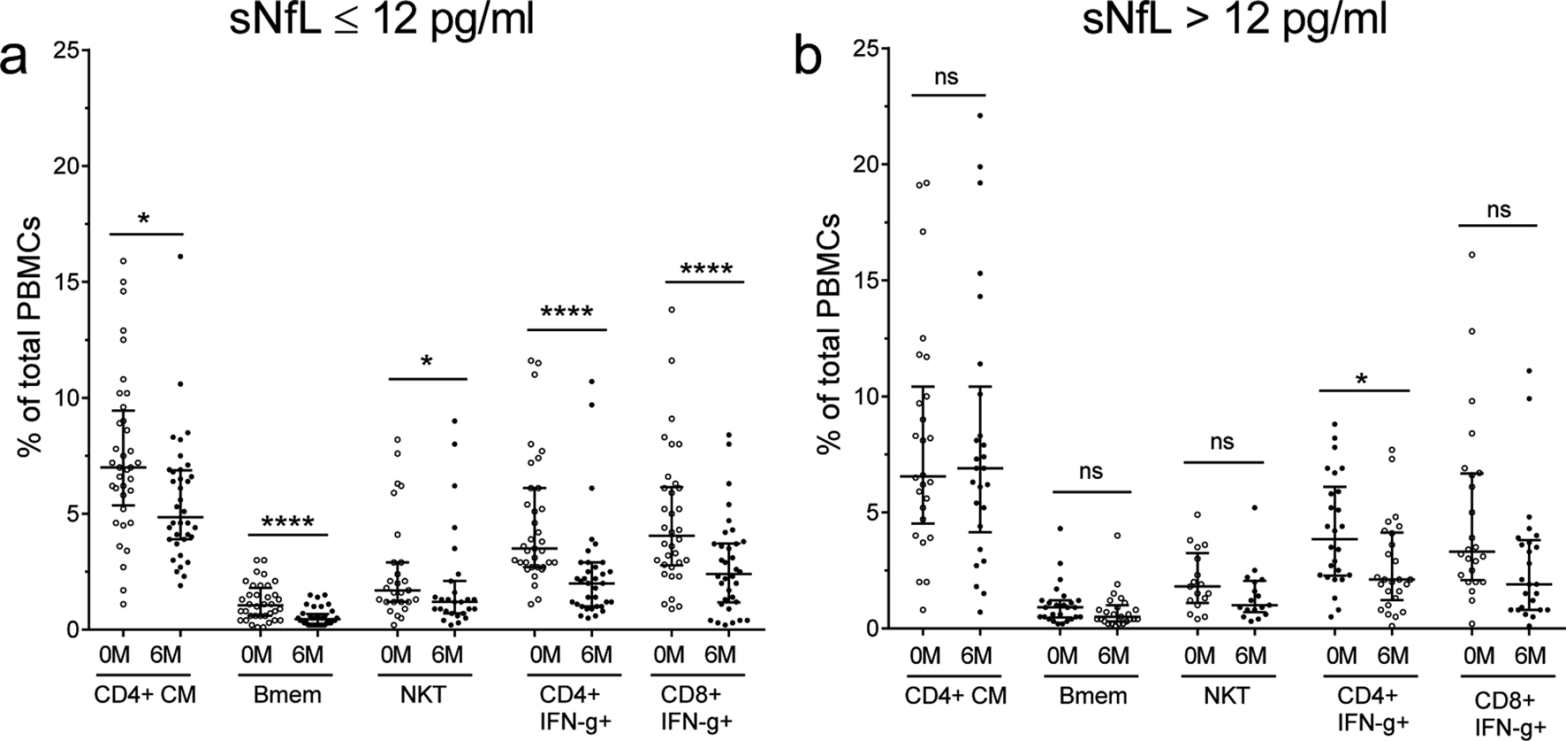
Blood cell subsets changes after 6 months of dimethyl fumarate (DMF) treatment depend on basal serum neurofilament light chain (sNfL) values. Percentages of blood Central Memory (CM) CD4+ T lymphocytes, memory B (Bmem) cells, Natural Killer T (NKT) cells, and CD4+ and CD8+ T lymphocytes producing Interferon-gamma (IFN-g) at baseline (0 M) and after six months (6 M) of dimethyl fumarate treatment in 62 relapsing–remitting multiple sclerosis patients showing baseline sNfL values ≤ 12 (n = 36, **a**) or > 12 (n = 26, **b**) pg/ml. Percentages are referred to total peripheral blood mononuclear cells (PBMCs). Medians and Interquartile Ranges are shown. ns: not significant. *p* values are corrected by Bonferroni test. *p < 0.05; **p < 0.01; ****p < 0.0001.

Since sNFL values below 12 pg/ml at baseline associated with NEDA status after a year of treatment, we aimed to further investigate if this cut-off value could also identify changes in any leukocyte subset. Thus, we explored changes in blood leukocyte populations after six months of DMF treatment in 62 RRMS patients with available peripheral blood mononuclear cells (PBMCs) samples, showing low (≤ 12 pg/ml, n = 36) or high (> 12 pg/ml, n = 26) baseline sNfL values. Both groups of patients showed significant decreases in effector memory CD4+ and CD8+ T lymphocytes, total and terminally differentiated CD8+ T lymphocytes, TNF-alpha+ CD8+ T lymphocytes and CD4+ T cells producing IFN-gamma. Results are shown in Supplementary Fig. S1. Remarkably, only patients with sNfL ≤ 12 pg/ml at baseline showed a decrease in the percentages of blood central memory CD4+ T lymphocytes (*p* = 0.02), memory B cells (*p* < 0.0001), Natural Killer T cells (*p* = 0.02) and CD8+ T lymphocytes producing IFN-gamma (*p* < 0.0001) after six months of DMF treatment (Fig. 4). No differences were observed after six months of DMF treatment in the remaining populations studied (Supplementary Fig. S1 and S2).

## Discussion

DMF has been shown to be an effective drug in the treatment of RRMS patients with intermediate disease activity^14^. However, some patients show a suboptimal response and need to escalate to a second-line therapy. Thus, it is highly important the early identification of optimal responders to DMF treatment, to avoid potential relapses or disability progression in suboptimal responders. Using ultra sensible SIMOA technique, we quantified sNfL values of 80 RRMS patients initiating DMF and at 3, 6 and 12 months of treatment, and studied their association with treatment response. Baseline sNfL results obtained in our cohort were similar to those reported in other series^12,13^. We explored differences between NEDA and ODA patients and found that the first group had lower sNfL values at baseline. These values allowed us to identify patients with high probability of being optimal responders 1 year after DMF treatment initiation. This is highly relevant, as achieving NEDA status after the first year of treatment associates with a high probability of remaining free of progression for longer periods^15^. sNfL values strongly associated with the presence of Gd+ enhancing lesions and are ultimately related to acute inflammation in MS^6,16–18^. Likewise, a higher proportion of ODA patients showed Gd+ enhancing lesions in baseline MRI in our cohort. However, differences were clearer when studying sNfL values at this time point. In consequence, they might be more accurate and accessible biomarkers compared to radiological variables for predicting treatment response in patients with moderate disease course, who have only limited radiological activity. To further explore the influence of previous disease activity in our results, we analyzed our data by logistic regression, correcting by the number of baseline Gd+ enhancing lesions and by NEDA status in the previous year of treatment initiation. We found that, although disease activity had an influence on baseline sNfL values, the ability of baseline sNfL values for identifying optimal responders to DMF treatment remained significant. Baseline sNfL levels also predicted the outcome of Fingolimod treatment^11^, which reinforces their value as a tool for personalized treatment in MS.

As described in various cohorts of MS patients treated with different disease modifying drugs^12,13^, we found that sNfL levels decreased during DMF treatment. The reduction did not depend on age, sex, previous treatments or disease duration and occurred in both NEDA and ODA patients, thus showing that sNFL decrease does not necessarily associate with an optimal response to the drug. However, reaching sNfL levels below 7 pg/ml after three months of DMF treatment allowed us to identify NEDA patients. These results suggest that early normalization of sNfL values are related with an optimal response. Thus, it is important to establish accurate reference values showing good inter-laboratory reproducibility. In this line, the use of the SIMOA platform gave excellent correlation across different laboratories^19^. The development of new highly sensitive platforms will represent a challenge to maintain sNFL standardization in the next future^20^.

We found that all patients experienced changes in their immunological profile at some degree after six months of DMF treatment. However, baseline sNfL values lower than 12 pg/ml implied deeper changes including significant decreases in some effector and memory populations previously associated with NEDA status^5,21–23^. This strongly suggests that these baseline sNfL values identify MS patients capable of modifying their abnormal immune response during DMF treatment and thus, with high probability of achieving an optimal response to the drug.

Although this data should be confirmed in multi-center studies, our results show that sNfL levels in MS patients are excellent biomarkers to early identify optimal responders to DMF.

## Patients and methods

### Patients

Eighty RRMS patients who initiated DMF treatment at the MS Unit of Ramon y Cajal University Hospital (Madrid, Spain) were consecutively included in the study.

Washout periods of at least one or two months were established for patients previously treated with first or second line drugs respectively.

### Patient follow-up

Patients were monitored at least every three months, with extra visits in case of relapses. Demographical, clinical and radiological variables recorded at baseline were listed in Table 1. In the follow-up visit after 12 months, patients were classified as follows: *optimal responders*, if they showed no evidence of disease activity (NEDA) in terms of absence of relapses, progression in the EDSS score or MRI activity, or *suboptimal responders,* if they showed evidence of ongoing disease activity (ODA). ODA status was defined by the presence of at least a relapse, or confirmed disability progression (CDP) measured with the EDSS score^24^, or new T2 or Gd+ enhancing lesions at 1 year MRI.

### Essential requirements of MRI used in this work

A MRI of the brain was essentially performed as described previously^5^ with the only difference being that slices of 3 mm thickness without gap between slices were acquired in the current study.

### Samples

Serum samples at baseline (immediately before), 3, 6 and 12 months after DMF treatment initiation were obtained from every patient and stored at -80ºC until used. Heparinized blood samples at baseline and at 6 months were also collected. PBMCs were obtained and cryopreserved as previously described^5^.

### sNfL detection

NfL values were quantified in 25 µl duplicate serum samples obtained from every patient by single molecule array (SIMOA) technique in a SR-X instrument (Quanterix, MA, USA), following manufacturer instructions. Baseline, 3, 6 and 12 months samples were assayed simultaneously to avoid inter-assay variability.

### Flow cytometry

Monoclonal antibodies used in this study are listed in Table 3. Extracellular protein labelling, PBMC stimulation and staining for intracellular cytokine detection was performed as described previously^5^. After antigen staining, scattered light properties and fluorescent emission by PBMCs were evaluated in a FACSCanto II flow cytometer (BD Biosciences). For establishing cut-off values of PBMC autoflorescence, isotype controls were employed. PBMCs subsets were identified by using FACSDiva Software V.8.0 (BD Biosciences) as previously reported^5^. At least 5 × 10^4^ events were analyzed. We used non-stimulated PBMCs as a control of basal intracellular cytokine production.

**Table 3:** Monoclonal antibodies (MoAb) used in the study.

MoAb	Manufacturer
CD8-FITC	Becton Dickinson
CD27-FITC	Becton Dickinson
IFN-gamma-FITC	Becton Dickinson
CD24-PE	Becton Dickinson
CD197(CCR7)-PE	Becton Dickinson
GM-CSF-PE	Becton Dickinson
IL-10-PE	Biolegend
CD3-PerCP	Becton Dickinson
CD38-PE-Cy5.5	Becton Dickinson
TNF-alpha-PerCP-Cy5.5	Becton Dickinson
CD19-PE-Cy7	Becton Dickinson
CD25-PE-Cy7	Becton Dickinson
CD45RO-APC	Becton Dickinson
CD56-APC	Becton Dickinson
IL-17-APC	R&D Systems
CD4-APC-H7	Becton Dickinson
CD8-APC-H7	Becton Dickinson
CD3-BV421	Becton Dickinson
CD127-BV421	Becton Dickinson
CD45-V500	Becton Dickinson

*GM-CSF* granulocyte/macrophage-colony stimulating factor, *IFN* interferon, *IL* interleukin, *TNF* tumor necrosis factor.

### Statistical analysis

We used GraphPad Prism 8.0 software (GraphPad Prism Inc, La Jolla, CA) and Stata 16 software (StataCorp LLC, Lakeway, TX) to perform statistical analyses. Absolute and relative frequencies were used to describe categorical variables, and mean and standard deviation or median and 25th–75th percentile (IQR) to illustrate continuous ones. Two-tailed Mann–Whitney U tests and two-tailed Wilcoxon matched-pair tests were employed to compare two independent groups of patients and to assess differences between baseline and 6 months samples respectively. Two-tailed Fisher's exact tests were used to analyze categorical variables, while Friedman ANOVA tests for paired data were utilized to compare sNfL results along treatment. Generalized Linear Model (GLM) tests were employed for testing interaction with age, sex, previous disease-modifying treatments or disease duration. Multivariate analyses were used for exploring influence of the presence of Gd+ enhancing lesions at baseline MRI study and of the NEDA status in the previous year in the ability of sNfL values for predicting NEDA at 12 months. sNfL cut-off values were established by using ROC curves; basal and 3-month sNfL values rendering the best specificity and sensibility were chosen. 95% confidence intervals were provided. *p* values were adjusted by using Bonferroni test. *p* values below 0.05 were assumed to be significant.

### Ethics declarations

This study was authorized by Clinical Research Ethics Committee of Ramón y Cajal University Hospital (approval number: 162-19). All experiments were conducted according to the Ramón y Cajal Health Research Institute (IRYCIS) guidelines on good scientific practice and to the Declaration of Helsinki. Every patient signed an informed consent and accepted the publication of the anonymized results before entering the study.

## Supplementary Information

Supplementary Legends.

Supplementary Information 1.[Replace ESM 2 with the attached "Supplementary_information_reviewed_ESM"].

## Data Availability

Anonymized data supporting the findings of this study will be shared by reasonable request from any qualified investigator during 3 years after the publication of the study.
