# Supplementary material for: Low serum neurofilament light chain values identify optimal responders to dimethyl fumarate in multiple sclerosis treatment
Source: Sci Rep. 2021 Apr 29;11:9299. doi: 10.1038/s41598-021-88624-7 (PMC8085019; doi:10.1038/s41598-021-88624-7)
Supplement: Supplementary file 1 — Supplementary Legends. [file 41598_2021_88624_MOESM1_ESM.docx]

**Supplementary Fig. S1**: Blood CD4+ and CD8+ lymphocyte subsets after 6 months of dimethyl fumarate (DMF) treatment depending on basal serum neurofilament light chain (sNfL) values. Percentages of blood CD4+ (a-d) and CD8+ (e-h) T lymphocyte subsets obtained at baseline (0M) and after six months (6M) of DMF treatment, in 62 relapsing-remitting multiple sclerosis patients showing baseline sNfL values ≤ 12 (n=36) or > 12 (n=26) pg/ml. Percentages are referred to total peripheral blood mononuclear cells (PBMCs). Medians and Interquartile Ranges are shown. P values are corrected by Bonferroni test. *: p<0.05; **: p<0.01; ****p<0.0001. CM: central memory; EM: effector memory; GM-CSF: granulocyte macrophage-colony stimulating factor; IFN-g: interferon-gamma; IL: interleukin; N: naïve; ns: not significant; T: total; TD: terminally differentiated; TNF-a: tumor necrosis factor-alpha; Treg: regulatory CD4+ T cells.

**Supplementary Fig. S2**: Blood B lymphocyte and NK cell subsets after 6 months of dimethyl fumarate (DMF) treatment depending on basal serum neurofilament light chain (sNfL) values. Percentages of blood B cell (a, b) and NK cell (c, d) subsets obtained at baseline (0M) and after six months (6M) of DMF treatment, in 62 relapsing-remitting multiple sclerosis patients showing baseline sNfL values ≤ 12 (n=36) or > 12 (n=26) pg/ml. Percentages are referred to total peripheral blood mononuclear cells (PBMCs). Medians and Interquartile Ranges are shown. P values are corrected by Bonferroni test. *: p<0.05; ****p<0.0001. Bmem: memory B cells; br: bright; GM-CSF: granulocyte macrophage-colony stimulating factor; NK: natural killer; ns: not significant; PB: plasmablasts; T: total; TNF-a: tumor necrosis factor-alpha.
