# Supplementary material for: Low serum neurofilament light chain values identify optimal responders to dimethyl fumarate in multiple sclerosis treatment
Source: Sci Rep. 2021 Apr 29;11:9299. doi: 10.1038/s41598-021-88624-7 (PMC8085019; doi:10.1038/s41598-021-88624-7)
Supplement: Supplementary file 2 — Supplementary Information 1.[Replace ESM 2 with the attached "Supplementary_information_reviewed_ESM"]. [file 41598_2021_88624_MOESM2_ESM.pdf]

LOW SERUM NEUROFILAMENT LIGHT CHAIN VALUES IDENTIFY OPTIMAL RESPONDERS TO DIMETHYL FUMARATE IN MULTIPLE SCLEROSIS TREATMENT

AUTHORS: Paulette Esperanza Walo-Delgado MD[1], Susana Sainz de la Maza MD[2], Noelia Villarrubia PhD[1*], Enric Monreal MD[2], Silvia Medina PhD[1], Mercedes Espiño PhD[1], José Ignacio Fernández-Velasco MSc[1], Eulalia Rodríguez-Martín PhD[1], Ernesto Roldán PhD[1], Daniel Lourido MD[3], Alfonso Muriel PhD[4], Jaime Masjuan-Vallejo MD[2], Lucienne Costa-Frossard MD[2], Luisa María Villar PhD[1]

AFFILIATIONS:

1. Immunology Department. Ramón y Cajal University Hospital. IRYCIS. REEM. Madrid. Spain.
2. Neurology Department. Ramón y Cajal University Hospital. IRYCIS. REEM Madrid. Spain.
3. Radiology Department. Ramón y Cajal University Hospital. IRYCIS. REEM Madrid. Spain.
4. Clinical Biostatistics Unit. Ramón y Cajal University Hospital. IRYCIS. University of Alcalá. CIBERESP. Madrid. Spain.

*CORRESPONDING AUTHOR:

Noelia Villarrubia
Department of Immunology, Hospital Universitario Ramón y Cajal
Ctra. Colmenar Km. 9.100, 28034, Madrid, Spain
E-mail address: noelia.villarrubia@salud.madrid.org
Telephone: +34913368795
Fax: +34913368809
ORCID: 0000-0002-2114-8632

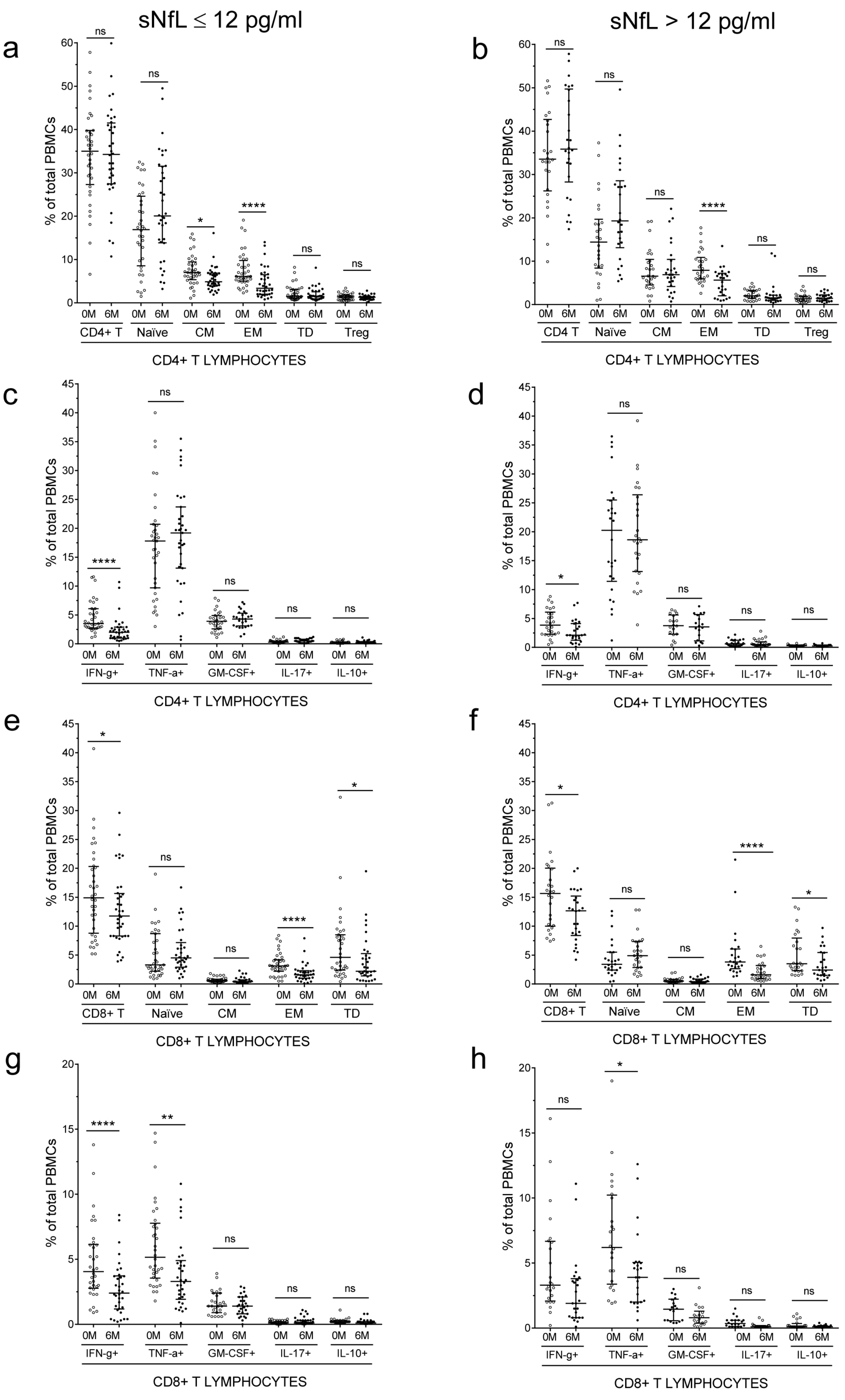

Supplementary Fig. S1

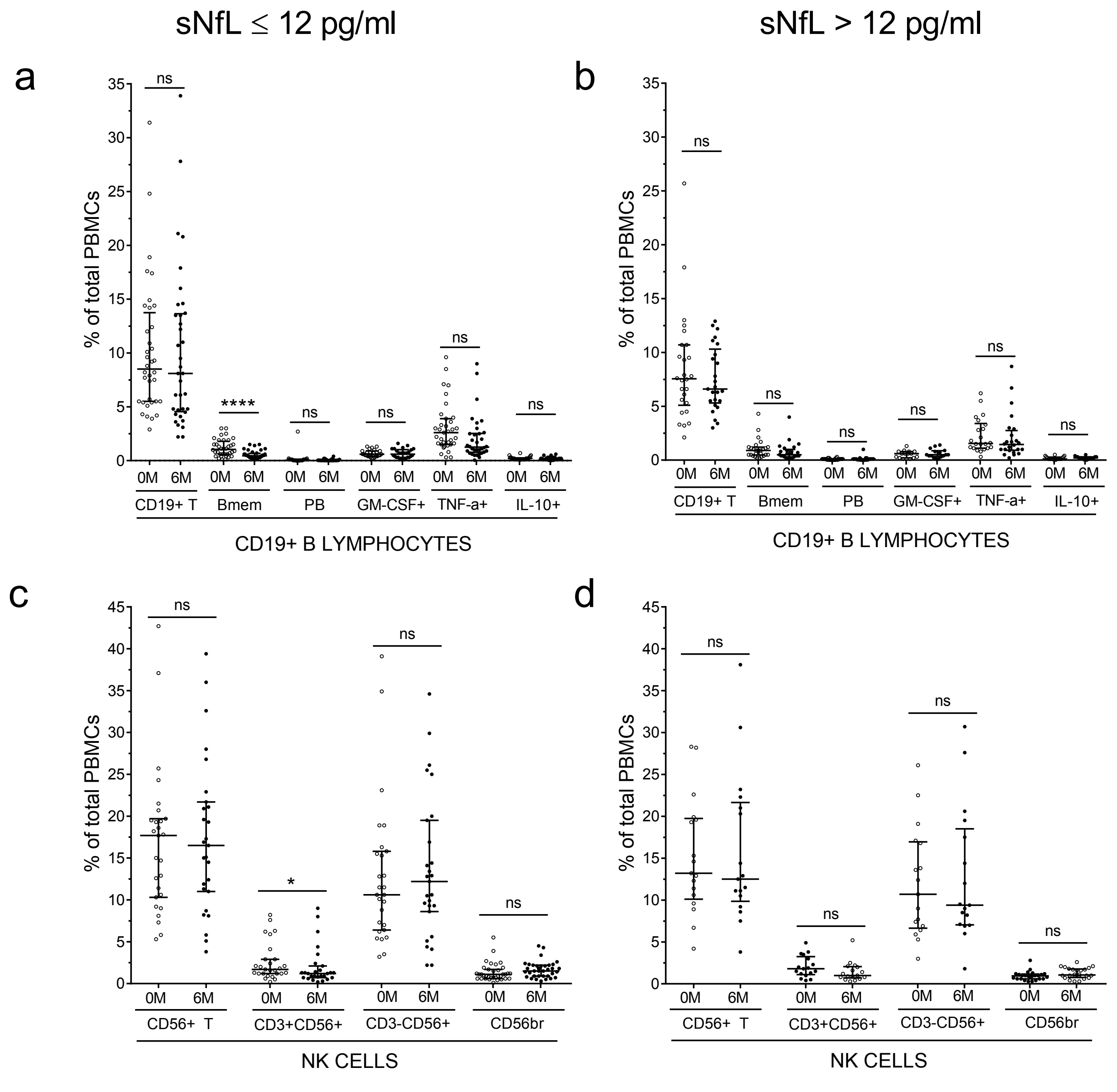

Supplementary Fig. S2
